# Intramuscular Mechanisms Mediating Adaptation to Low-Carbohydrate, High-Fat Diets during Exercise Training

**DOI:** 10.3390/nu12092496

**Published:** 2020-08-19

**Authors:** Emily E. Howard, Lee M. Margolis

**Affiliations:** 1 Military Nutrition Division, U.S. Army Research Institute of Environmental Medicine, Natick, MA 01760, USA; emily.e.howard14.ctr@mail.mil; 2 Oak Ridge Institute for Science and Education, Oak Ridge, TN 37830, USA

**Keywords:** fat oxidation, substrate utilization, PDH activity, CPT-1, fat adaptation

## Abstract

Interest in low-carbohydrate, high-fat (LCHF) diets has increased over recent decades given the theorized benefit of associated intramuscular adaptations and shifts in fuel utilization on endurance exercise performance. Consuming a LCHF diet during exercise training increases the availability of fat (i.e., intramuscular triglyceride stores; plasma free fatty acids) and decreases muscle glycogen stores. These changes in substrate availability increase reliance on fat oxidation for energy production while simultaneously decreasing reliance on carbohydrate oxidation for fuel during submaximal exercise. LCHF diet-mediated changes in substrate oxidation remain even after endogenous or exogenous carbohydrate availability is increased, suggesting that the adaptive response driving changes in fat and carbohydrate oxidation lies within the muscle and persists even when the macronutrient content of the diet is altered. This narrative review explores the intramuscular adaptations underlying increases in fat oxidation and decreases in carbohydrate oxidation with LCHF feeding. The possible effects of LCHF diets on protein metabolism and post-exercise muscle remodeling are also considered.

## 1. Introduction

Interest in implementing low-carbohydrate, high-fat (LCHF) diets during exercise training has persisted over recent decades given the associated increase in fat oxidation and reduction in carbohydrate oxidation during exercise [1,2,3]. Lower rates of carbohydrate oxidation have been attributed to the ‘sparing’ of muscle glycogen stores [4], which may delay fatigue and improve endurance performance [5,6]. Advantageous shifts in substrate oxidation and the potential to improve performance has led athletes and coaches to incorporate continuous or periodized LCHF diets into training [4,7].

Adaptations in whole-body substrate oxidation with LCHF feeding during exercise training may be attributed, in part, to alterations in substrate availability, as intramuscular triglyceride (IMTG) stores increase and muscle glycogen concentrations decrease [8,9,10]. Changes in dietary intake and substrate availability modulate the activity and abundance of key protein related to substrate metabolism, resulting in intramuscular adaptations that drive changes in substrate oxidation at rest and during exercise. LCHF feeding increases reliance on fat as a fuel source, and decreases muscle glycogenolysis during prolonged aerobic exercise [4,11]. These changes in substrate utilization are robust and remain even after endogenous or exogenous carbohydrate availability is increased [12,13,14,15].

Given the continued interest in LCHF diets, a comprehensive understanding of mechanisms driving shifts in fuel utilization during exercise may facilitate an appropriate and specific application of these dietary strategies and an understanding of what individuals, if any, may benefit. Therefore, this narrative review considers the intramuscular adaptations to LCHF feeding during exercise training underlying associated decreases in carbohydrate oxidation and increases in fat oxidation.

## 2. Substrate Utilization during Submaximal Exercise

Sustaining contractile activity during aerobic exercise relies on the degradation of carbohydrate, fat and, to a limited extent, protein to maintain a supply of reducing equivalents for adenosine triphosphate (ATP) production. Endogenous sources of oxidized carbohydrate include muscle glycogen and blood glucose (via liver glycogenonlysis and gluconeogenesis), while fat oxidation is primarily maintained by intramuscular triglycerides (IMTGs) and adipose tissue-derived plasma free fatty acids (FFAs). Amino acid oxidation likely contributes <5% of total ATP production [16], which may be increased under conditions of low carbohydrate availability [17]. Oxidation of branched chain amino acids (BCAAs; leucine, isoleucine, and valine), in particular, occurs in skeletal muscle and is enhanced with exercise [18].

The proportion of fat versus carbohydrate fuel sources oxidized by working muscle is largely dictated by the intensity and duration of exercise [19,20]. The oxidation of plasma FFAs sustains energy production during low-intensity exercise (~25% of maximal O_2_ uptake [VO_2max_]), while muscle glycogen, blood glucose, and IMTGs are utilized to a greater extent as exercise intensity increases [19]. Maximal fat oxidation typically occurs between 45 and 65% of VO_2max_ [21,22], with the oxidation of carbohydrate (primarily muscle glycogen) predominating at exercise intensities exceeding that threshold [19]. During exercise of longer duration, energy production increasingly relies on plasma FFA and blood glucose, and the contribution of IMTGs and glycogen progressively declines [19,23].

## 3. Fat Adaptation

Muscle fatigue and compromised performance during prolonged exercise coincide with the depletion of muscle and liver glycogen stores and the associated decrease in carbohydrate oxidation [5,6]. Strategies for delaying muscle fatigue and maximizing performance have therefore focused on slowing the rate of muscle and liver glycogen utilization. This is primarily achieved through endurance training itself, which results in increased rates of fat oxidation and sparing of carbohydrate stores during submaximal exercise [24]. Efforts to enhance this adaptive response have often focused on dietary intake since the proportion of fuel derived from carbohydrate versus fat at rest and during exercise is influenced by substrate availability [25,26]. Typical nutritional strategies include increasing muscle glycogen stores prior to an event (i.e., increasing abundance of a limited fuel) [27] or providing exogenous carbohydrate during exercise to maintain carbohydrate availability [28,29]. Alternatively, there has been continued interest in increasing availability and reliance on fat (i.e., plasma FFA, IMTGs) as a fuel source to spare limited muscle glycogen stores during prolonged exercise and delay the onset of fatigue [1,30]. Potential strategies have included both acute and chronic modifications to dietary fat intake.

**Figure 1:**
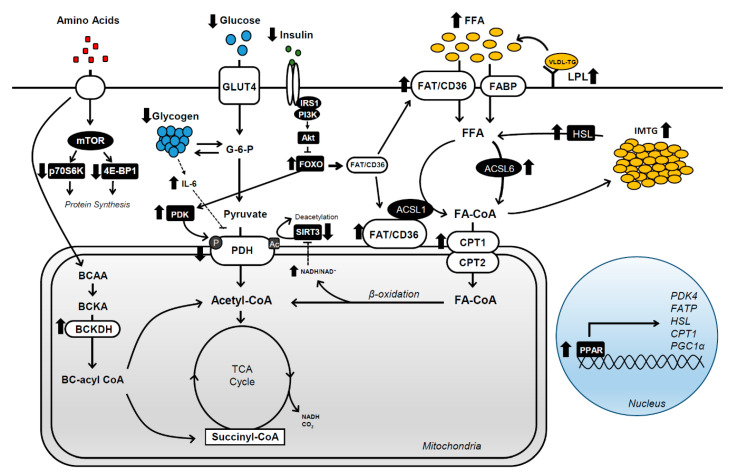
Overview of intramuscular adaptations driving the increase in fat oxidation and decrease in carbohydrate oxidation during exercise with low-carbohydrate, high-fat (LCHF) diets. 4E-BP1, 4E binding protein 1; ACSL1/6, long-chain acyl-coA synthetase 1/6; BCAA, branched chain amino acid; BC-acyl CoA, branched chain acyl-CoA; BCKA, branched chain α-keto acid; BCKADH, branched chain α-keto acid dehydrogenase; CPT1/2, carnitine palmitoyl transferase 1/2; FA-CoA, fatty acyl-CoA; FAT/CD36; fatty acid translocase; FATP; fatty acid transport protein; FFA, free fatty acid; FOXO, fork head box O; HSL, hormone-sensitive lipase; IL-6, interleukin-6; IRS1, insulin receptor substrate 1; LPL, lipoprotein lipase; mTOR, mammalian target of rapamycin; p70S6K1, p70 ribosomal protein S6 kinase; PDH, pyruvate dehydrogenase; PDK, pyruvate dehydrogenase kinase; PGC1α, peroxisome proliferator activated receptors gamma coactivator 1α; PI3K, phosphatidylinositol 3-kinase; SIRT3, sirtuin-3; VLDL-TG, very low-density lipoprotein-triglyceride.

Acutely increasing FFA availability by consuming a high-fat meal before a prolonged exercise bout has limited effects on patterns of substrate oxidation and performance outcomes [31,32]. In contrast, consuming a LCHF diet (>60% energy intake from fat, <20% energy intake from carbohydrate) during exercise training elevates rates of fat oxidation and decreases carbohydrate oxidation by reducing muscle glycogen utilization during submaximal exercise [11,33]. This method of ‘fat adaptation’ while aerobically training increases FFA availability (i.e., IMTGs and plasma FFAs) and decreases muscle glycogen stores [11,34,35], resulting in the activation and abundance of key proteins involved in substrate metabolism (Figure 1). These shifts in substrate availability and oxidation can occur within 5 days of initiating LCHF feeding [33,36].

There has been additional interest in ‘dietary periodization’ strategies that involve short-term LCHF feeding (5–14 days) followed by periods of carbohydrate loading in relation to specific training sessions or competition. These interventions allow for muscle adaptation to high-fat feeding, while lessening the negative effects of low glycogen stores on performance by restoring glycogen to normal levels. Interestingly, LCHF diet-mediated changes in substrate utilization are sustained following glycogen restoration and the provision of exogenous carbohydrate [4,12,13,14,15]. For example, rates of fat oxidation were greater and carbohydrate utilization was lower during exercise after five days of a LCHF diet and carbohydrate restoration (i.e., one day of high-carbohydrate feeding and the provision of exogenous carbohydrate prior to the exercise trial) versus an isoenergetic high-carbohydrate control diet [13]. These findings suggest the adaptive response driving changes in substrate utilization with LCHF feeding persists even when macronutrient content of the diet is altered.

Furthermore, insightful work by Leckey et al. [37] compared LCHF (~18% carbohydrate and ~68% fat) versus low-carbohydrate, high-protein (HPRO; ~19% carbohydrate, 67% protein) diets during 5 days of exercise training. Fat oxidation rates increased and carbohydrate oxidation decreased during exercise in LCHF and HPRO compared to pre-diet values, a change that was likely due to lower pre-exercise muscle glycogen content and elevated plasma FFAs in both groups. However, rates of fat and carbohydrate oxidation were higher and lower, respectively, in LCHF versus HPRO, suggesting adaptations to LCHF diets are not driven solely by low-carbohydrate availability, but by alterations specific to increased dietary fat intake (i.e., elevated IMTG concentrations). Interestingly, changes in substrate oxidation during exercise persisted in LCHF but not HPRO after one day of CHO restoration and the provision of carbohydrate before exercise testing [37]. These findings suggest that it is the high-fat, not the low-carbohydrate component, of the LCHF diet driving molecular adaptations and concomitant alterations in substrate utilization that persist with increases in endogenous and exogenous carbohydrate availability.

While intramuscular adaptations to LCHF diets with or without carbohydrate restoration may theoretically benefit exercise capacity by enhancing fat utilization and sparing muscle glycogen stores, it is important to note that a consistent, favorable effect on endurance-based performance outcomes has not been demonstrated. Although a few studies report performance benefits [11,38], many show no significant changes [4,12,13,39] and even performance decrements [40,41]. It has also been suggested that performance benefits may be limited to specific individuals (i.e., responders vs. non-responders) [1]. Given the interest in high-fat feeding that persists despite limited and conflicting evidence on physical performance, more human research is needed to understand under what conditions, if any, an individual may benefit from such interventions. A comprehensive understanding of intracellular mechanisms underlying the adaptive response is also necessary to optimize potential interventions and facilitate appropriate and specific applications.

## 4. Carbohydrate Metabolism

Intramuscular mechanisms underlying the LCHF diet-driven decreases in carbohydrate oxidation would be expected to involve key regulatory sites of carbohydrate metabolism. This regulation typically occurs at the level of blood glucose uptake, breakdown of muscle glycogen stores, glycolysis, and the generation of acetyl-coA for the TCA cycle. Glucose is transported into the cell following the activation and translocation of glucose transporter type 4 (GLUT4) to the plasma membrane by insulin or exercise, and is subsequently trapped after phosphorylation by hexokinase. Additional sites of regulation include glycogen phosphorylase and phosphofructokinase (PFK), which catalyze the rate-limiting steps in glycogenolysis and glycolysis, respectively. The rate of carbohydrate oxidation is also dependent on pyruvate dehydrogenase (PDH), which catalyzes the irreversible decarboxylation of pyruvate to acetyl-coA. This critical step controls the delivery of carbohydrate-derived substrate to the mitochondria for complete oxidation.

### 4.1. Glucose Transport

Glucose transport into the cell does not appear to be altered by short-term LCHF diets. Work from our laboratory showed that consuming a LCHF (16% carbohydrate, 72% fat) versus high-carbohydrate (65% carbohydrate, 25% fat) diet for 24 h after performing an exhaustive exercise bout to elicit low or adequate glycogen stores, respectively, did not alter exogenous carbohydrate oxidation during 80 min of steady-state exercise while consuming a carbohydrate drink (95 g glucose and 51 g fructose; 1.8 g/min) [42]. These findings were supported by a similar expression of GLUT4 and hexokinase mRNA before and after the exercise bout in both groups [42]. Burke et al. [4] similarly showed that plasma-derived glucose disposal was unchanged during exercise after 5 days of a LCHF (19% carbohydrate, 68% fat) versus high-carbohydrate (74% carbohydrate, 13% fat) diet and one day of high-carbohydrate feeding to restore muscle glycogen concentrations. These findings suggest that reductions in carbohydrate oxidation with LCHF feeding can be attributed to a sparing of muscle glycogen. GLUT4 protein content also remained unchanged with short-term (5 days) fat adaptation and carbohydrate restoration [15]. These findings are important when considering additional performance-enhancing nutritional strategies, such as the provision of exogenous carbohydrate during exercise. This work suggests that despite declines in the delivery of carbohydrate-derived substrates to the mitochondria with short-term LCHF feeding [14], these interventions may still be appropriate in fat-adapted individuals since glucose transport into the cell and exogenous carbohydrate oxidation is not impaired under these conditions. 

In contrast, impairments in glucose transport may contribute to reduced capacity for carbohydrate oxidation following long-term LCHF diets. Seven weeks of a LCHF (21% carbohydrate, 62% fat) versus high-carbohydrate (65% carbohydrate, 20% fat) diet during exercise training in previously untrained men, followed by one week of carbohydrate restoration, impaired leg glucose uptake in fat-adapted participants [43]. Although levels of GLUT4 were similar between groups and could not account for the change in muscle glucose uptake, the possibility exists that GLUT4 translocation was altered [43]. In cyclists habituated to >6 months of LCHF ketogenic diets (~50 g/day of carbohydrate), protein content of GLUT4 and its upstream regulator insulin receptor substrate 1 (IRS1) was lower compared to cyclist habitually consuming mixed-macronutrient diets (~272–561 g/day of carbohydrate) [44]. Lower GLUT4 and IRS1 in cyclists consuming LCHF versus mixed-macronutrient diets likely contributed to observed reductions in rates of glucose clearance during an oral glucose tolerance test (OGTT) under resting condition. These findings suggest reductions in proteins regulating glucose uptake may be apparent after LCHF diets of longer duration, and may reflect adaptation to chronic reductions in glucose availability [44].

### 4.2. Glycogenolysis

While reductions in carbohydrate oxidation during submaximal exercise following LCHF diets have been attributed to a sparing of muscle glycogen stores [4], it is not entirely clear whether changes in glycogen phosphorylase (PHOS; rate limiting enzyme in glycogenolysis) activity contribute to this effect. Some insight comes from exercise bouts performed in the presence of artificially elevated FFA. Exercising at high intensities (80% VO_2max_) with concomitant infusion of Intralipid-heparin solution to increase plasma FFA concentrations resulted in significant sparing of muscle glycogen in a subset of subjects [45]. While the percentage of active PHOS remained unchanged, it was speculated that inhibition occurred given declines in allosteric activators (AMP and ADP) and decreased substrate availability (inorganic phosphate [Pi]) [45]. Reduced flux through phosphorylase at lower power outputs (40 and 65% VO_2max_) with artificially increased plasma FFA was also attributed to decreased AMP accumulation [46]. These lower accumulations of AMP and ADP under high-fat conditions may result from increased availability of NADH and ATP from fat oxidation at the onset of exercise [47]. Whether similar mechanisms extend to LCHF diet conditions is unclear, as decreased glycogenolysis at the beginning of exercise in fat-adapted individuals occurred independent of changes in ADP and AMP abundance [14].

Reductions in glycogenolysis following LCHF diets may largely result from lower glycogen stores at the initiation of exercise. Consuming a LCHF versus mixed-macronutrient diet for short (5 days), moderate (6 weeks), and long-term (>6 months) durations reduced fasted/resting glycogen content by ~50% and lowered glycogenolysis during exercise [11,34,35]. Consuming a LCHF diet may also impair post-exercise glycogen recovery. Mice consuming LCHF (23% carbohydrate, 57% fat) versus standard (60% carbohydrate, 13% fat) diets for three days prior to an endurance exercise bout, had lower muscle glycogen concentrations from pre-exercise to 120 min post-exercise, despite adequate amounts of glucose provided during the post-exercise recovery period [48]. These findings collectively suggest that the reduced use of glycogen for fuel during exercise following LCHF feeding is likely the result of lower content rather than diminished PHOS activity.

### 4.3. PDH Activity

Rates of carbohydrate oxidation are largely controlled by PDH activity, which is attenuated at rest and during exercise following LCHF feeding [42,49,50]. These LCHF diet-mediated decreases in PDH activity may impair the use of available carbohydrate for energy production during high-intensity exercise. Estimated muscle glycogenolysis and PDH activity were decreased during one min of sprint cycling (150% peak power output) at the end of a 20 min steady-state ride (70% VO_2peak_) in individuals adapted to five days of LCHF (~18% carbohydrate, ~67% fat) versus high-carbohydrate (~70% carbohydrate, ~15% fat) feeding plus one day of carbohydrate loading [14]. These findings suggest that LCHF diet-mediated decreases in PDH activity and the associated delivery of carbohydrate-derived substrates to the mitochondria persist even at exercise intensities that maximally activate PDH. This may translate to decreases in high-intensity exercise performance, as six days of LCHF feeding (68% fat) followed by 1 day of carbohydrate loading (8–10 g carbohydrate/kg) decreased average power output during a high-intensity 1-km sprint (>90% W_peak_) compared to a high-carbohydrate control trial (68% carbohydrate) [40].

PDH is inhibited and stimulated by PDH kinase (PDK; phosphorylation) and PDH phosphatase (PDP; dephosphorylation), respectively. While decreases in resting PDH activation with LCHF diets have been attributed to increases in PDK activity [51,52], it is unclear if similar regulation extends to exercise conditions. PDH activity increases rapidly at the onset of exercise due to acute accumulations of intramuscular calcium and pyruvate that stimulate PDP and inhibit PDK, respectively [53]. Reduced activation of PDH with LCHF diets appears to be mediated by alterations in PDK activity, as the pharmacological inhibition of PDK was shown to attenuate decreases in PDH activity before and during 60 min of cycling after 3 days of a LCHF diet (10% carbohydrate, 75% fat) [50].

LCHF diet-related increases in resting PDK activity have been attributed to elevated PDK4 protein content and possible stable adaptations or covalent modifications [51,52,54]. Increases in PDK4 abundance may result from greater FFA-mediated activation of peroxisome proliferator-activated receptor (PPAR) transcription factors, which regulate PDK4 gene expression in vitro [55]. The forkhead box O1 (FOXO1) transcription factor may also modulate PDK4 abundance by binding to the promoter region of the PDK4 gene [56]. Cell culture models have shown FOXO1 protein content is sensitive to increased fatty acid availability. Specifically, FOXO1 protein content was increased in C2C12 myotubes following incubation with long chain fatty acids [57]. LCHF diets may also enhance FOXO1 transcriptional activity given the reduction in carbohydrate intake and associated decrease in insulin-stimulated inhibition of FOXO1 via IRS-1/phosphatidylinositol 3-kinase (PI3K)/Akt signaling [58]. These potential mechanisms of PDK4 regulation are supported by work in humans showing increased resting FOXO1, PPARα, PPARγ, and PDK4 mRNA after 3 days of LCHF feeding (10% carbohydrate, 75% fat) versus an isocaloric, mixed-macronutrient control diet (55% carbohydrate, 30% fat) [50]. Expression of FOXO1 and PPARα were also associated with PDK4 expression at rest and during exercise [50].

Additional regulation of PDH activity during LCHF feeding may occur through mitochondrial NAD^+^-dependent sirtuin 3 (SIRT3) activity. It has been hypothesized that increases in fat oxidation due to elevated FFA availability would result in a greater abundance of mitochondrial NADH at rest and during the onset of exercise [47,59]. A higher NADH to NAD+ ratio may attenuate SIRT3 activity, given NAD^+^ is a cofactor for SIRT3-mediated deacetylation [60]. Loss of SIRT3 activity in vitro results in hyperacetylation and suppression of PDH, and induces a switch from carbohydrate oxidation towards fatty acid utilization [61]. Whether PDH acetylation is altered in human skeletal muscle following a LCHF diet remains to be determined.

LCHF diet-related decreases in PDH activity during exercise may also be driven by lower muscle glycogen stores and increased interleukin (IL)-6 signaling. Muscle glycogen levels are decreased when consuming a LCHF diet during exercise training [35]. Commencing exercise with depleted muscle glycogen stores increases production and release of IL-6 from skeletal muscle during exercise [62,63]. Muscle-specific IL-6 knockout in rats increases PDH activity at rest and during exercise [64,65], suggesting that IL-6 may inhibit PDH activity. While these findings suggest that low glycogen-related increases in IL-6 production during exercise may suppress PDH activity, this potential mechanism of regulation may not account for LCHF diet-related decreases in PDH activity that persist with the restoration of muscle glycogen stores and increased carbohydrate availability [14].

## 5. Fat Metabolism

LCHF diets increase the utilization of fatty acids for energy production at rest and during submaximal exercise. Whether this adaptive response is the result of enhanced muscle uptake of fat or increased utilization of elevated IMTG stores is not fully understood. Some insight is provided by Zderic et al. [8], who showed that increased rates of fat oxidation during exercise following LCHF feeding (24% carbohydrate, 60% fat) persisted despite the pharmacological inhibition of adipose tissue lipolysis and a resulting decrease in plasma FFA [8]. Rates of fatty acid appearance and plasma-derived fatty acid oxidation were also unchanged at rest and during exercise after seven days of a LCHF diet (25% carbohydrate, 60% fat) in young men [66]. These findings indicate plasma FFA have a limited role in mediating the increase in fat oxidation with LCHF diets. Conversely, there are several reports that LCHF diets increase plasma FFA availability and uptake [67,68,69]. While potential reasons for this discrepancy include differences in the duration of diets, the degree of carbohydrate restriction (i.e., <5% vs. >5% carbohydrate), or the macronutrient content of the final pre-exercise meal [8], these findings suggest additional work is necessary to understand the role of plasma FFA in the adaptive response to LCHF feeding.

Increased fat oxidation with LCHF diets may be driven by greater utilization of fatty acids derived from triglycerides (i.e., from very low-density lipoprotein-triglyceride (VLDL-TG) or IMTG) for energy production. TG-derived fatty acid oxidation tended to be higher at rest and was significantly higher during exercise after 7 days of LCHF feeding [66]. Helge et al. [67] also showed enhanced VLDL-TG uptake during exercise after seven weeks of a LCHF (21% carbohydrate, 62% fat) versus high-carbohydrate (65% carbohydrate, 20% fat) diet during exercise training. IMTG levels are also increased after short and long-term LCHF diets with or without carbohydrate loading [8,15,70,71], and are used during exercise in proportion to starting concentrations [72]. Specifically, IMTG stores were increased by ~36% after only 2 days of LCHF (24% carbohydrate, 60% fat) versus high-carbohydrate (65% carbohydrate, 22% fat) feeding in endurance-trained cyclists [8]. These findings suggest that altered substrate storage (i.e., increased IMTG) and an enhanced uptake of VLDL-TG contribute to increases in fat oxidation during exercise with LCHF feeding.

### 5.1. Fatty Acid Uptake

Increases in VLDL-TG oxidation with LCHF diets may be driven by changes in lipoprotein lipase (LPL) activity. LPL facilitates fatty acid release from VLDL-TG and its activity is increased in muscle after 4 weeks of a LCHF diet (29% carbohydrate, 54% fat) [70]. Increases in fat oxidation may also be regulated at the level of FFA uptake into the muscle, which involves several transport proteins (e.g., membrane-associated fatty acid binding protein (FABP), fatty acid transport protein 1 and 4 (FATP1,4), and fatty acid translocase (FAT)/CD36). Levels of FAT/CD36 protein are associated with estimated rates of whole-body fat oxidation during 1 h of endurance exercise in rats [73], suggesting increases in FAT/CD36 protein content may be involved in the adaptive response to LCHF feeding. Twelve weeks of a LCHF diet (20% carbohydrate, 60% fat) plus endurance training in rats elevated FAT/CD36 mRNA compared to a LCHF diet alone [74]. In humans, one day of LCHF (16% carbohydrate, 72% fat) versus high-carbohydrate (65% carbohydrate, 25% fat) feeding after an exhaustive exercise bout to deplete muscle glycogen increased FAT/CD36 and FABP mRNA expression at rest and after 80 min of steady-state exercise [42]. Five days of LCHF (<20% carbohydrate, >65% fat) versus high-carbohydrate (70–75% carbohydrate, <15% fat) feeding in well-trained cyclists similarly increased resting FAT/CD36 mRNA and protein content, though resting FABP protein content was unchanged in these individuals [26]. FAT/CD36 protein content was also unaltered after five days of LCHF feeding with carbohydrate restoration [15,37]. These data may suggest that dietary periodization negates adaptive increases in fatty acid transporter content. However, there was no assessment of FAT/CD36 following the five days of LCHF feeding only, so it is unclear if translational adaptations occurred in these participants prior to carbohydrate restoration [15,37].

Along with abundance, the cellular location of FAT/CD36 may be important in its contribution to increased fat oxidation. However, changes in cellular location and related effects are not fully understood. FAT/CD36 translocates from an intracellular pool to the muscle membrane in response to exercise, insulin, and other stimuli [75]. However, it is unknown if FAT/CD36 translocation to the muscle membrane is altered with LCHF feeding, as only changes in gene expression and total protein content have been measured [26,42]. FAT/CD36 also resides on the outer mitochondrial membrane, where it facilitates the delivery of long-chain fatty acid to the mitochondria during exercise [76]. Whether elevated FAT/CD36 protein content is indicative of enhanced muscle uptake of fatty acids or an increase in their delivery to the mitochondria is therefore unclear. Future work is necessary to determine if increases in FAT/CD36 are similarly or differentially distributed between the muscle and mitochondrial membrane with LCHF feeding.

The upstream regulation of FAT/CD36 translocation to the muscle membrane and LPL activity may occur through the FOXO1 transcription factor, which is activated in the presence of increased fatty acid availability in cultured muscle cells [57]. Activated FOXO1 promotes FAT/CD36 recruitment to the muscle membrane [77] and the induction of LPL in vitro [78]. Interestingly, the up-regulation of FAT/CD36, and the related influx of fatty acids, reinforces muscle reliance on fatty acid utilization through the downstream activation of PPARδ/β, which increases FOXO1 and PDK4 expression (i.e., suppresses carbohydrate oxidation) [57]. This positive feedback regulation of FOXO1 activity provides a potential mechanism by which chronic changes in muscle FAT/CD36 content could promote long-term shifts in muscle fuel preference [77].

### 5.2. IMTG Storage and Breakdown

Fatty acids transported into the cell are catalyzed into their active form, fatty acyl-CoA, by long-chain acyl-CoA synthetase (ACSL), and are subsequently used for IMTG synthesis or β-oxidation. These divergent metabolic fates of intracellular fatty acids are determined by isoform-specific expression of ACSL. ACSL6, for example, likely facilitates lipid storage, as its gene expression increases acutely following a high-fat meal in both rodents and humans, and its knockdown in cultured muscle cells reduces cellular triacylglycerol content [79]. ACSL6 mRNA expression was increased in rats consuming a LCHF diet (20% carbohydrate, 60% fat) with or without aerobic exercise training versus sedentary rats consuming a high-carbohydrate diet (70% carbohydrate, 10% fat) [80]. Changes in ACSL6 protein abundance were also positively associated with intramyocellular lipid content [80]. The possibility exists that ACSL6 up-regulation under LCHF diet conditions during exercise training mediates increases in IMTG stores.

While the breakdown of elevated IMTG stores may contribute to enhanced fatty acid oxidation during exercise with LCHF feeding [8,66], regulatory mechanisms have not been fully elucidated. The primary lipolytic enzymes in skeletal muscle include adipose triacylglycerol lipase (ATGL) and hormone-sensitive lipase (HSL), which regulate complete TG hydrolysis. Interestingly, enhanced lipolytic enzyme content may occur independent of IMTG stores. Despite higher IMTG content, mice fed a LCHF diet had lower HSL protein content than mice fed a control diet [81]. However, when mice consuming a LCHF were exercise trained for 8 weeks ATGL and HSL protein content were higher, despite IMTG content being lower or the same as mice consuming LCHF or control diets, respectively [81]. These data indicate to improve sensitivity in IMTG lipolysis, LCHF diets need to be coupled with exercise training to stimulate molecular adaptations in lipolytic enzyme content.

### 5.3. Mitochondrial Fatty Acid Transport

ACSL1-mediated conversion of fatty acids to fatty acyl-CoAs directs a subset of plasma and IMTG-derived fatty acids to the mitochondria for oxidation and ATP production. Rats subjected to 12 weeks of LCHF feeding and exercise training versus 12 weeks of low-fat feeding and no exercise had increases in ACSL1 gene expression, but not protein content [80]. Fatty acyl-CoAs generated by ACSL1 are subsequently transported into the mitochondria for β-oxidation via action of the rate-limiting enzyme carnitine palmitoyl transferase 1 (CPT1) at the outer mitochondrial membrane. LCHF diet-mediated increases in FAT/CD36 may enhance this process, as the interaction of FAT/CD36 and ACSL at the mitochondrial membrane has been suggested to regulate fatty acyl-CoA availability to CPT1 [76]. Skeletal muscle CPT1 activity was also increased over baseline levels at day 10 and day 15 of a LCHF diet (19% carbohydrate, 69% fat) in endurance-trained cyclists [33]. CPT1 gene expression was unchanged after 5 days of LCHF feeding, however, indicating that changes in CPT-1 activity may be regulated by posttranslational mechanisms [26].

### 5.4. PPARs

Peroxisome proliferator activated receptors (PPARα/δ) are central regulators of fatty acid oxidation [82]. PPARα and PPARδ are nuclear receptors that target and activate genes that increase fatty acid oxidation [82]. Expression of these PPARs is increased in response to LCHF diets and exercise training [83,84]. In cell culture and mouse models, the pharmacological overexpression of PPARδ increases FATP, HSL, CPT1, hydroxyacyl-CoA dehydrogenase (HADHA), and long-chain acyl-CoA dehydrogenase (LCAD) expression in skeletal muscle [85]. Up-regulation of these genes governing fatty acid metabolism resulted in greater fat oxidation [85]. Similarly, elevations in circulating FFA following consumtion of LCHF diets in rat models results in increased expression of PPARα, and binding of PPARδ to the mCPT1 promoter [84]. Increases in PPARs also appear to contribute to an increased capacity to oxidize fat due to increased mitochondrial enzymes involved in fatty acid oxidation, the citrate cycle, and respiratory chain [84]. Results from cell and animal experiments have been translated to humans, as expression of PPARα was increased following 5 days of LCHF feeding in lean individuals without exercise [86]. Along with the up-regulation in PPARα, expression of PPAR gamma coactivator 1α (PGC1α) and PDK4 were also increased. PGC1α is the well-described transcription factor that functions as the central regulator of mitochondrial biogenesis [87]. Increased expression of PGC1α is associated with higher rates of ꞵ-oxidation and reductions in glycolysis potentially through feed-forward activation of PPARα and PPARδ [88,89]. As described above, PPARs play an integral role in the regulation of PDK4 to inhibit glycolytic flux [90]. Together these data highlight the integral role of PPARs in increasing fat oxidation and inhibiting glucose oxidation following LCHF diets. Important to note, up-regulation of PPARs in response to LCHF feeding may be unique to lean individuals, with no change or down-regulation observed in obese individuals [86]. Divergent responses in PPAR expression based on BMI may indicate that lean individuals have a higher degree of metabolic flexibility, enabling the adaptation to fuel use based on substrate availability [86].

## 6. Protein Metabolism

Amino acid metabolism accounts for a limited portion of energy production during endurance exercise (~5%) [16]. Branched-chain amino acids (BCAAs; leucine, isoleucine, valine), in particular, are mobilized for oxidation by working muscle [91]. BCAAs are transaminated to their keto-acids via branched-chain aminotransferase (BCAT), and subsequently oxidized by the rate-limiting enzyme branched-chain α-keto acid dehydrogenase (BCKDH). This irreversible reaction generates acyl-CoA derivatives that are converted into the TCA cycle intermediates acetyl-CoA or succinyl-CoA. Exercise enhances BCKDH activity to facilitate this response [92].

While changes in carbohydrate and fat oxidation with LCHF diets are well-established, the effect of these dietary strategies on protein metabolism during exercise is relatively unknown. Some insight comes from work evaluating the effect of low muscle glycogen content on exercise-induced amino acid oxidation. Amino acid oxidation is greater when exercising in a glycogen-depleted state compared to adequate glycogen stores, as evidenced by indirect measures of protein turnover [17,93]. Findings are similar when evaluating whole body net protein balance and skeletal muscle protein turnover during exercise in men who consumed a low-carbohydrate (low glycogen) or high-carbohydrate (adequate glycogen) diet for 2 days after an exhaustive exercise bout [94]. Exercising with low muscle glycogen concentrations increased leucine oxidation and attenuated protein synthesis during exercise compared to adequate glycogen stores [94]. An efflux of amino acids from skeletal muscle under low glycogen conditions also suggests that protein catabolism was increased to provide substrates for energy yielding oxidative metabolism [94]. These findings collectively suggest that exercise-induced increases in amino acid oxidation may be heightened by LCHF diets resulting in low muscle glycogen content.

Recent work from Gillen et al. [95] indicates the oxidative protein losses from exercise performed with low carbohydrate availability may increase dietary protein requirements. Participants in this study performed high-intensity interval training (HIIT) in the evening, followed by a 10-km run the next morning. Carbohydrate availability was kept low (LOW) in one trial by providing most of the daily carbohydrate intake prior to the HIIT session and withholding carbohydrate post-exercise and overnight. In contrast, carbohydrate was consumed before and after the HIIT, and prior to the 10-km run in the high-carbohydrate availability (HIGH) trial. Performing exercise with low carbohydrate availability increased phenylalanine oxidation during 8 h of post-exercise recovery [95]. This translated to a ~0.12 g/kg/d difference in protein requirements between HIGH and LOW. While this difference is relatively small, it is important in the context of ensuring adequate protein consumption to replace oxidative losses of amino acids, and to augment training-induced skeletal muscle remodeling and physiological adaptations. Given the decreases in muscle glycogen stores that occur when consuming a LCHF diet while exercise training, dietary protein requirements may be increased slightly under these conditions to facilitate post-exercise recovery. It should be noted that these differences were the result of lower glycogen availability and not due to increased fat intake. Whether LCHF diets alter amino acid oxidation and post-exercise protein requirements has not been determined.

The impact of chronic LCHF feeding on pathways regulating muscle protein metabolism are unclear. Some insight is provided by work examining anabolic signaling responses to exercise following acute changes in muscle glycogen content and free fatty acid availability. Recent work by Knudsen et al. [96], for example, evaluated the anabolic response to a resistance exercise protocol in mice with or without manipulating glycogen stores. Pharmacologically increasing muscle glycogen content over baseline levels enhanced contraction-stimulated phosphorylation and activation of ribosomal protein S6 kinase 1 (p70S6K1) and ribosomal protein S6 (rpS6), two downstream targets of mTOR. However, whether mTOR-p70S6k1-rpS6 signaling is attenuated under low muscle glycogen versus normal glycogen conditions and after an endurance exercise bout is unclear.

Consuming a high-fat meal immediately after exercise during a LCHF diet may also influence the post-exercise anabolic response. Acutely increasing free fatty acid availability through intravenous lipid infusion blunted the muscle protein synthetic response to ingestion of protein (21 g amino acids) in healthy young men [97]. This was associated with a complete suppression of muscle 4E binding protein 1 (4E-BP1) phosphorylation, a downstream target of mTOR-mediated anabolic signaling [97]. Hammond et al. [98] similarly examined intracellular mediators of protein synthesis following a morning bout of high-intensity interval training and afternoon steady-state running under LCHF (2.5 g/kg carbohydrate, 3.5 g/kg fat) or high-carbohydrate (10 g/kg carbohydrate, 0.8 g/kg fat) dietary conditions. LCHF versus high-carbohydrate feeding tended to suppress the protein translational regulator p70S6K1(*P* = 0.08) three hours post steady-state exercise despite sufficient protein intake [98]. These findings collectively suggest high-fat feeding immediately after exercise may blunt the muscle protein synthetic response and post-exercise muscle remodeling, highlighting the importance of meal timing when consuming a LCHF diet. Whether the acute changes in signaling observed under low glycogen or high free fatty acid conditions result in adaptations to pathways regulating protein metabolism or associated changes in muscle size during chronic consumption of LCHF diets is unclear.

## 7. Conclusions

Chronic alterations in substrate availability while consuming a LCHF diet during exercise training enhances the activation and abundance of several proteins involved in substrate metabolism. These intramuscular adaptations drive increases in fat oxidation and decreases in carbohydrate utilization during submaximal exercise, and persist even when carbohydrate availability (endogenous or exogenous) is increased. Adaptations underlying increased rates of fat oxidation with LCHF feeding include increased transport capacity, increased storage and breakdown of IMTGs, and heightened CPT1-mediated mitochondrial fatty acid delivery. Decreased rates of carbohydrate oxidation are likely due to attenuated glycogenolysis and PDH activity.

Interestingly, exogenous carbohydrate oxidation is not impaired with LCHF feeding, suggesting that this is still a viable fueling strategy during prolonged exercise in fat-adapted individuals since glucose transport is not compromised. Individuals interested in consuming a LCHF diet must also consider daily protein intake and the timing of high fat meals, as exercising with low muscle glycogen stores may increase daily protein requirements, and consuming a high-fat meal after exercise may blunt the post-exercise protein synthetic response and muscle remodeling.
